# Morphometry and food preference in relation to sex and hematological values of Eurasian collared dove (*Streptotella decaocto*)

**DOI:** 10.5455/javar.2023.j675

**Published:** 2023-06-30

**Authors:** Hasnain Akmal, Shabbir Ahmad, Iqra Akram, Muqadas Shahzadi, Sajid Ali, Alllah Dita, Arva Mehmood, Nadeem Bukhash, Irfan Ahmad, Taqi Shahid Jaffari, Khurram Shahzad

**Affiliations:** Department of Zoology, University of Okara, Okara, Pakistan

**Keywords:** Eurasian collared dove, hematology, feeding habit, morphometric parameters

## Abstract

**Objective:**

The study investigated the gut content and recorded morphometric and hematological parameters in the Eurasian collared dove (*Streptopelia decaocto*).

**Materials and Methods:**

24 samples of healthy birds (12 from each sex) were collected from different wetlands in Punjab, Pakistan, from December 2022 until February 2023. Birds were captured live for blood samples, morphometric, and gut analyses.

**Results:**

The current study revealed that mensural measurements showed no significant differences in all parameters except tail length, which was significantly longer in males (14.59 ± 0.30) compared to females (12.88 ± 0.43). Hematological parameters were hemoglobin, 23.95 gm/dl; red blood cells, 3.97 × 10^6^/μl; white blood cells, 429.9.67 × 10^3^/μl; hematocrit, 72.14%; mean corpuscular volume, 183.24 FL; mean corpuscular hemoglobin, 61.70 pg; mean corpuscular hemoglobin concentration, 32.37 pg; platelets, 7.01/μl; and red cell distribution width, 110.86/μl. The percentages of neutrophils, lymphocytes, monocytes, and eosinophils were 71.33%, 23.03%, 3.30%, and 1.43%, respectively. The gut content of the Eurasian collared dove mainly consisted of rice, wheat, corn, and millet seeds. Some stony materials were also present.

**Conclusion:**

Our study concluded that male and female Eurasian collared doves are alike in biometrics (except tail length) and hematological profiles. Gut content and weight were also similar. Males were slightly larger than females. The gut content showed that the Eurasian collared dove mainly feeds on rice, wheat, corn, and millet seeds. In this study, hematological parameters were also studied.

## Introduction

The Eurasian collared dove was first described by a Hungarian naturalist, Imre Frivaldszky, in 1838 with the scientific name *Columba decaocto* [1]. Later, this bird was placed in the genus *Streptopelia* by French ornithologist Bonaparte [2]. *Columba decaocto* belongs to the Columbidae, the family of pigeons and doves [3]. The Eurasian collared dove exists worldwide, including Europe, Asia Minor, Turkistan, North China, Japan, Palestine, Iraq, Iran, and Great Britain [4]. Its range extends to India, Pakistan, and Ceylon, while only post-monsoon visitors visit the Nepal valley. The bird mainly nests in forests of babool, dhak, and similar trees but avoids moist evergreen tracts [5].

Morphometry is the quantitative analysis of morphological structures, including counting, size, and shape. Morphometric analytical parameters are valuable for assessing the impact of ecological, developmental, genetic, and mutational factors or changes on the shape and size of organisms [6]. It can measure a trait of significance for evolution and detect changes in shape to derive something from its ontogeny, function, or evolutionary relationships. The main goal was to test hypotheses statistically on factors influencing the form. Using morphological data is a growing technique for predicting resource consumption and niche organization in animal groups. Only species that are sufficiently distinct morphologically from one another can coexist in animal populations because varied morphological adaptations should reflect disparities in resource usage [7]. Both males and females were similar throughout the year [8].

The study of hematological parameters provides information about animal nutrition, physiology, and pathological status [9]. The values of different hematological parameters could be a source of diagnosis for many diseases [10].

The Eurasian collared dove is highly granivorous. They are often seen in large flocks in cultivated areas, picking up seeds from the floor of freshly harvested crops, but they also feed on bushes and trees as barriers [11]. They mainly feed on millet, corn, rice, sunflower, wheat, and invertebrates. The Eurasian collared dove can store much food and water in its crop to reduce the danger in open areas [12]. Therefore, this study aimed to compare a few morphometric parameters and feeding preferences and evaluate hematological parameters in breeding pairs of Eurasian collared doves.

## Materials and Methods

### Ethical approval

All procedures carried out on the animals in this study followed the rules set by the University of Okara’s Ethical Committee (UO/ERC/2023/32A).

### Study area

12 pairs of *S. decaocto* were captured using mist nets from the cultivable fields of districts Bahawalnagar and Okara. These regions are semi-dried and have a winter temperature range of 16°C–26°C and 15°C–25°C, respectively. The sampling started in December 2022 and continued until February 2023.

### Blood sample analysis

After capture, the birds were anesthetized with ketamine hydrochloric acid (10 mg/kg) and diazepam (0.2 mg/kg). 5 μl blood was taken in an ethylenediamine tetraacetic acid tube from the jugular vein of each anesthetized bird. An automatic hematological analyzer was used for the analysis of hematological parameters. The recorded hematological parameters included red blood cell (RBC) counts, red cell distribution width (RDW), white blood cell (WBC) counts, hemoglobin concentration, platelets (PLT), platelet distribution width, packed cell volume, Mean corpuscular hemoglobin (MCH), Mean corpuscular volume (MCV), Mean corpuscular hemoglobin concentration (MCHC), Mean platelet volume, monocytes, lymphocytes, neutrophils, and eosinophils.

### Morphometric parameters

After taking blood samples, all anesthetized birds were subjected to morphometric measurements. Body weight was taken using an electronic balance (0.001 gm). Body length, wingspan, wing length, length of the longest primary feather, tail length, tarsus, central toe length, chest circumference, bill size, and head size were recorded with the help of a measuring tape and scale, according to Aslam et al. [13] and Zafar et al. [14].

### Food preference

After taking blood samples and morphometry, each anesthetized bird was dissected. The gastrointestinal tract from each sample was separately packed in polythene bags and labeled with sex, Collector’s name, Field no., sample no., and date. After labeling, gastrointestinal tracks were placed in an icebox and transported to the ornithological lab. Each stomach was dissected for content removal and then washed with standard process testing sieves. A dissecting microscope (IRMECO model SESYG306; 60X) was used to identify gut contents. Gut contents were separated and identified using available diagrams and the discretion of seeds [15].

**Table 1.** Hematological parameters of Eurasian collared dove collected from different wetlands of Punjab, Pakistan.

Variable	SE	Mean ± SD
HGB (gm/dl)	0.52	23.95 ± 0.89
WBC (×10^3^/μl)	3.18	429.9 ± 5.51
RBC (×10^6^/μl)	0.007	3.97 ± 0.02
HCT (%)	0.32	72.14 ± 0.56
MCV (FL)	0.34	183.24 ± 0.60
MCH (pg)	0.21	61.70 ± 0.36
MCHC (gm/dl)	0.40	32.37 ± 0.69
PLT (×103/μl)	0.02	7.01 ± 0.04
RDW (FL)	0.34	110.86 ± 0.58
Neutrophils	0.23	71.33 ± 0.40
Lymphocytes	0.13	23.03 ± 0.23
Monocytes	0.16	3.30 ± 0.28
Eosinophil’s	0.04	1.43 ± 0.07

SD = Standard deviation; SE = Standard error.

**Figure 1.**
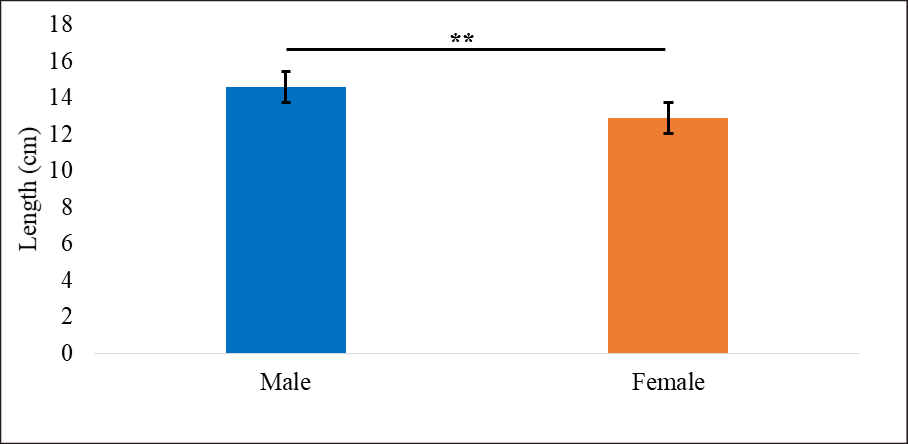
Comparison of tail length of both sexes *S. decaocto* (*p < 0.05; **p < 0.01).

### Statistical analysis

Data were analyzed through a standard statistical method (mean, standard error of mean, and range) using IBM SPSS (v. 21). The significance of the difference was tested using an unpaired *t*-test at 0.05 levels.

## Results and Discussion

### Hematology

Hematological parameters are used for diagnosing and evaluating disease therapy in birds and mammals [16]. This also provides health status information and information about immunology. It also provides information about parasitic infections and the toxicity caused by various toxicants. The present study offers physiological reference values for adult birds of this species (Table 1). All hematological parameters were higher than [17]. The values of hematological parameters in the current study were also higher than [18], except for lymphocytes (49.93 ± 0.14) and eosinophils (1.87 ± 0.53). This was a naval study of the hematological parameters of the Eurasian collared dove that can be used as a reference in future research (Table 1).

### Morphometry

The most common and easiest way to find out the difference between both sexes is morphometry. It also gives information about the size, age, and status of the bird. Morphometry describes the birds’ geological separation of the same species and sex differences [6]. A significant difference was observed in the tail length of both sexes (Fig. 1), but no other morphometric parameters showed any significant differences (Table 2).

Both sexes are almost indistinguishable, as reported by Snow et al. [19]. Our outcomes were similar to those of Campbell and Walters [20] in the case of body length and those of Cramp and Brooks [21] in the case of wingspan and body weight. However, no significant difference was observed between the head sizes of males and females, which contradicts the study [8] that found a significant difference in the head sizes of male (4.74 cm) and female (4.58 cm) Eurasian collared doves. The difference might be due to the age and size of the samples. The other reason could be the changing environment.

**Table 2.** Comparison of biometrics of male and female Eurasian collared dove.

Characters	Gender *n* = 12 each	Mean ± SD	Range	SE	*p*-value
Body weight (g)	Female	157.6 ± 4.63	143.8–173.0	3.004	0.9142^ns^
	Male	158.1 ± 2.13	144.0–172.2	2.45	
Body length (cm)	Female	31.84 ± 0.86	29.7–35.0	0.45	0.7672^ns^
	Male	31.64 ± 0.74	29.7–34.8	1.97	
Wingspan (cm)	Female	48.22 ± 1.91	46.3–50.7	0.40	0.5386^ns^
	Male	48.56 ± 1.70	47.2–51	0.34	
Wing length (cm)	Female	21.71 ± 0.74	21–23	0.17	0.8205^ns^
	Male	21.76 ± 1.07	21.2–22.6	0.13	
Longest primary feather (cm)	Female	15.99 ± 0.79	14.3–17.0	0.23	0.5091^ns^
	Male	16.27 ± 0.98	14.3–18.2	0.33	
Tarsus (cm)	Female	2.592 ± 0.18	2.0–2.9	0.07	0.0809^ns^
	Male	2.400 ± 0.05	1.9–2.9	0.07	
Central toe length (cm)	Female	3.092 ± 0.20	2.2–3.7	0.15	0.3634^ns^
	Male	2.900 ± 0.15	1.8–3.4	0.13	
Tail length (cm)	Female	12.88 ± 0.43	11.8–14.1	0.24	0.0002**
	Male	14.59 ± 0.30	13–16.6	0.29	
Chest circumference (cm)	Female	19.89 ± 0.53	18.3–22.4	0.31	13.8^ns^
	Male	20.36 ± 0.31	18.8–22	0.32	
Bill length (cm)	Female	2.392 ± 0.32	2.0–3.3	0.11	0.5779^ns^
	Male	2.292 ± 0.26	1.5–3.2	0.13	
Head size (cm)	Female	5.300 ± 0.36	4.8–6.1	0.13	0.7251^ns^
	Male	5.192 ± 0.26	3.7–6.7	0.27	

** = Highly significant difference (*p *< 0.01).

NS = Non-significant (*p *> 0.05); SD = Standard deviation; SE = Standard error.

**Table 3.** Comparison of gut contents in male and female Eurasain collared dove.

Character	Gender *N* = 12 for each	Mean	SD	SE	*t*-value	*p*-value
Total weight of stomach (gm)	Male	7.395	0.502	0.251	1.64	0.24^ns^
	Female	7.618	0.845	0.423		
Weight of food material (gm)	Male	1.829	0.518	0.259	1.52	0.27^ns^
	Female	2.072	0.310	0.155		
Weight of empty stomach (gm)	Male	5.678	0.183	0.092	1.86	0.20^ns^
	Female	5.546	0.584	0.292		

NS = Non-significant (*p *> 0.05); SD = Standard deviation; SE = Standard error.

**Table 4.** Gut content of male and female *S. decaocto*.

Types of food	Weight of different gut content (%)		
	Male	Female	*p*-value
Rice	31	30	0.68^ns^
Stone	30	28	o.61^ns^
Wheat	7	6	0.77^ns^
Millet	20	18	0.82^ns^
Plant Materail	4	8	0.23^ns^
Corn	7	10	0.57^ns^

NS = Nonsignificant difference (*p*-value > 0.05).

Lower body weight (male 158.1 gm, female 157.6 gm) was observed as compared to Varga and Juhász [22] and Salazar-Borunda et al. [8]. However, our results were similar to those of Varga and Juhász [22] and Salazar-Borunda et al. [8], as no significant difference was observed in wing length.

The tail of the male Eurasian collared dove was significantly longer (14.59 ± 0.30 cm) than the female (12.88 ± 0.43 cm). So, in this way, our study differed from Salazar-Borunda et al. [8]. Chest circumference, length of the longest primary feather, and central toe length have not been studied previously and are described for the first time in the present study.

### Food preferences

There was no significant difference in the total weight of the stomach, weight of food material, or weight of empty stomachs for both males and females (Table 3). The gut analysis showed that *S. decaocto *is highly granivorous and feeds mainly on rice, wheat, corn, and millet seeds (Table 4). These kinds of feeding habits were also previously recorded by Poling and Hayslette [23]. Similar food habits were also observed by Eddajjani et al. [24], who found that the Eurasian collared dove mainly feeds on cultivated grains, i.e., barley, rice, wheat, linseed, and mustard, with a small proportion of weeds. El-Mansi et al. [11] and Ali and Ripley [25] also supported our results, as they found that these birds feed on seeds and grains that include paddy grains, mustard, linseed, wheat, and various weeds.

## Conclusion

Our study concluded that male and female Eurasian collared doves are alike in biometrics (except tail length) and hematological profiles. Gut content and weight were also similar. Males were slightly larger than females. The gut content showed that the Eurasian collared dove mainly feeds on rice, wheat, corn, and millet seeds. In this study, hematological parameters were also studied.

## List of Abbreviations

MCH, Mean corpuscular hemoglobin; MCHC, Mean corpuscular hematocrit; MCV, Mean corpuscular volume; PLT, Platelets; RBC, Red blood cells; RDW, Red cell distribution, WBC, White blood cells.
